# Supplementary figures and images for: Micro-biopsy for detection of gene expression changes in ischemic swine myocardium: A pilot study
Source: PLoS One. 2021 Apr 28;16(4):e0250582. doi: 10.1371/journal.pone.0250582 (PMC8081259; doi:10.1371/journal.pone.0250582)

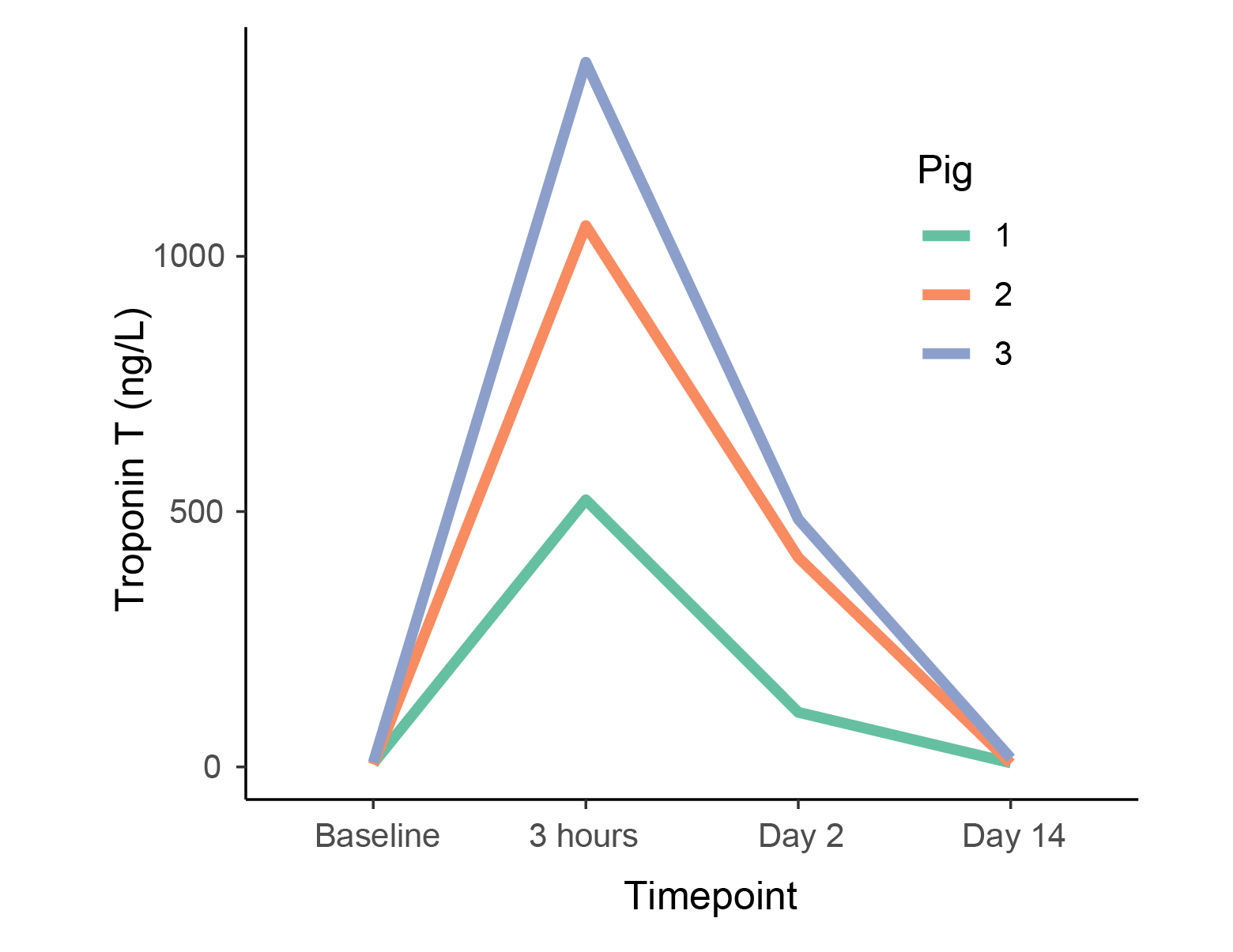

Supplement: S1 Fig — Troponin-T assays from venous blood drawn at different timepoints show a distinct surge in all swine (n = 3) at three hours after left anterior descending artery occlusion, indicating myocardial injury. (TIF) [file pone.0250582.s001.tif]

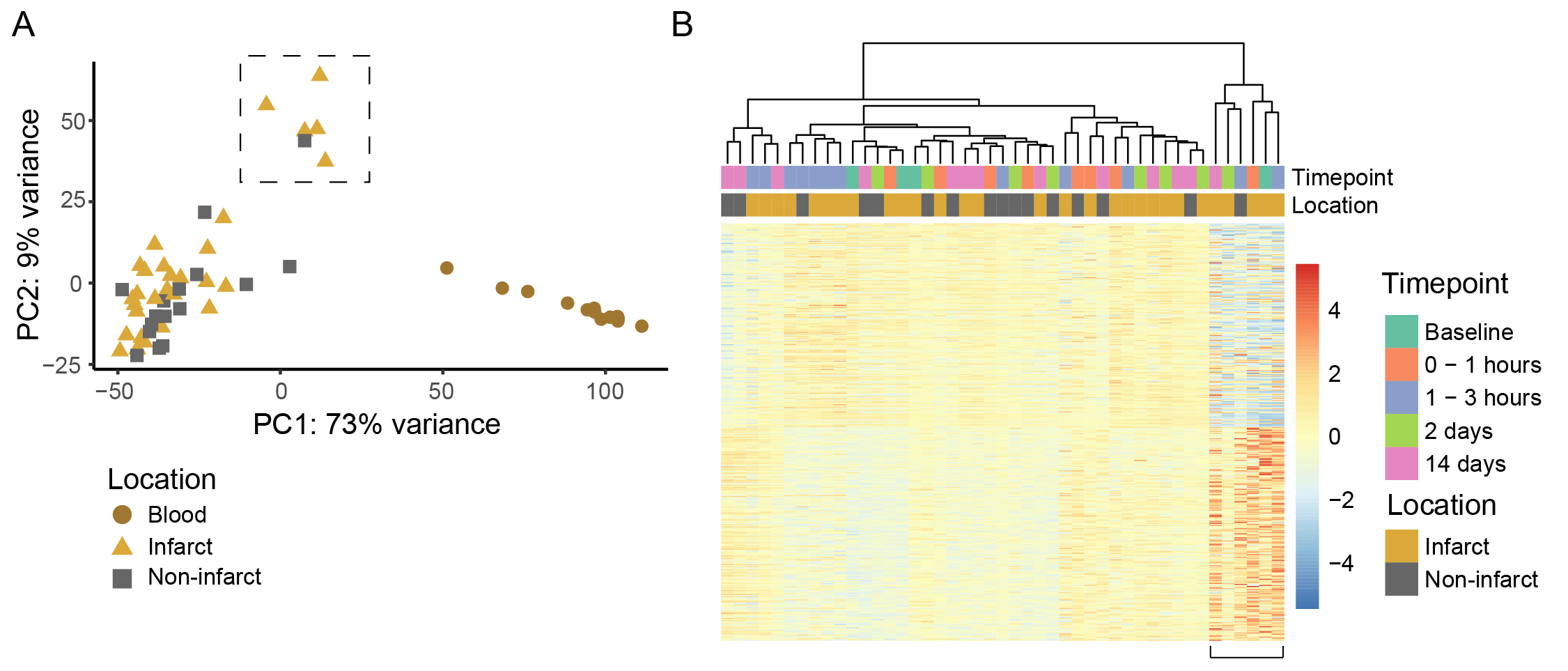

Supplement: S2 Fig — (A) PCA plot of top 500 high variance genes on all samples after excluding technically failed samples (n = 59). The plot shows good separation between blood and myocardial samples, apart from outlier samples marked by a dashed rectangle (n = 6). (B) Heatmap of normalized expression of genes up-regulated in heart samples (n = 198, top rows) and the outlier samples (n = 206, bottom rows). All heart samples were included (n = 45). Outlier samples indicated in (A) constitute a separate cluster (bottom bracket). (TIF) [file pone.0250582.s002.tif]
